# A new mouse model of GLUT1 deficiency syndrome exhibits abnormal sleep-wake patterns and alterations of glucose kinetics in the brain

**DOI:** 10.1242/dmm.038828

**Published:** 2019-09-01

**Authors:** Tamio Furuse, Hiroshi Mizuma, Yuuki Hirose, Tomoko Kushida, Ikuko Yamada, Ikuo Miura, Hiroshi Masuya, Hiromasa Funato, Masashi Yanagisawa, Hirotaka Onoe, Shigeharu Wakana

**Affiliations:** 1 Japan Mouse Clinic, RIKEN BioResource Research Center, Tsukuba, Ibaraki 305-0074, Japan; 2 Laboratory for Pathophysiological and Health Science, RIKEN Center for Biosystems Dynamics Research, Kobe, Hyogo 650-0047, Japan; 3 International Institute for Integrative Sleep Medicine (WPI-IIIS), University of Tsukuba, Ibaraki 305-8575, Japan; 4 Resource Advancement Unit, Integrated Bioresource Information Division, RIKEN BioResource Research Center, Tsukuba, Ibaraki 305-0074, Japan; 5 Department of Anatomy, School of Medicine, Faculty of Medicine, Toho University, Tokyo 143-8540, Japan; 6 Life Science Center for Survival Dynamics (TARA), University of Tsukuba, Ibaraki 305-8575, Japan; 7 Department of Molecular Genetics, University of Texas Southwestern Medical Center, Dallas, TX 75390, USA; 8 Human Brain Research Center, Kyoto University Graduate School of Medicine, Kyoto 606-8501, Japan

**Keywords:** ENU mutagenesis, Epilepsy, GLUT1DS, Glucose transporter 1

## Abstract

Dysfunction of glucose transporter 1 (GLUT1) proteins causes infantile epilepsy, which is designated as a GLUT1 deficiency syndrome (GLUT1DS; OMIM #606777). Patients with GLUT1DS display varied clinical phenotypes, such as infantile seizures, ataxia, severe mental retardation with learning disabilities, delayed development, hypoglycorrhachia, and other varied symptoms. *Glut1^Rgsc200^* mutant mice mutagenized with N-ethyl-N-nitrosourea (ENU) carry a missense mutation in the *Glut1* gene that results in amino acid substitution at the 324th residue of the GLUT1 protein. In this study, these mutants exhibited various phenotypes, including embryonic lethality of homozygotes, a decreased cerebrospinal-fluid glucose value, deficits in contextual learning, a reduction in body size, seizure-like behavior and abnormal electroencephalogram (EEG) patterns. During EEG recording, the abnormality occurred spontaneously, whereas the seizure-like phenotypes were not observed at the same time. In sleep-wake analysis using EEG recording, heterozygotes exhibited a longer duration of wake times and shorter duration of non-rapid eye movement (NREM) sleep time. The shortened period of NREM sleep and prolonged duration of the wake period may resemble the sleep disturbances commonly observed in patients with GLUT1DS and other epilepsy disorders. Interestingly, an *in vivo* kinetic analysis of glucose utilization by positron emission tomography with 2-deoxy-2-[fluorine-18]fluoro-D-glucose imaging revealed that glucose transportation was reduced, whereas hexokinase activity and glucose metabolism were enhanced. These results indicate that a *Glut1^Rgsc200^* mutant is a useful tool for elucidating the molecular mechanisms of GLUT1DS.

This article has an associated First Person interview with the joint first authors of the paper.

## Abstract (precis)

**Summary:** New phenotypes are revealed by a *GLUT1* deficiency mutant mouse model carrying a missense mutation in *Glut1*.

## INTRODUCTION

Glucose is one of the major nutritional components of food that provides energy to the brain by traversing through glucose transporter 1 (GLUT1) proteins, which act as exclusive transporters that carry glucose through the blood-brain barrier (BBB). A dysfunction of the GLUT1 protein causes infantile epilepsy, which is designated as GLUT1 deficiency syndrome (GLUT1DS; OMIM #606777). GLUT1DS, characterized by an autosomal-dominant heredity, has the following clinical phenotypes: infantile seizures, ataxia, learning disability with severe mental retardation, delayed development and hypoglycorrhachia, among others (Pearson et al., 2013; De Vivo et al., 1991).

The first case report of GLUT1DS was published in 1991 (De Vivo et al., 1991), and the link between a mutation in the *GLUT1* gene and the symptoms of GLUT1DS was demonstrated in 1998 (Seidner et al., 1998). Traditional anticonvulsants are not effective for GLUT1DS, but seizures are suppressed by treatment with a ketogenic diet (Klepper et al., 2004). Ketone bodies are transported by monocarboxylate transporters (MCTs) and pass through the BBB. Under conditions of ketosis, the ketone bodies are consumed as an alternative energy source for glucose in the brain. Zonisamide, an antiepileptic drug that does not inhibit glucose uptake into erythrocytes *in vitro*, was effective for controlling seizures in two GLUT1DS patients (Takahashi et al., 2008). Additionally, lithium-chloride treatment significantly increased glucose uptake into skin fibroblasts derived from control and GLUT1DS patients (Ullner et al., 2009). These findings suggest that drug therapy could be an alternative to a ketogenic diet for GLUT1DS. However, in order to develop new drugs or therapeutic methods, animal models of GLUT1DS are necessary.

In a previous study, heterozygous *Glut1*-gene knockout (KO) mice showed epileptiform discharges on electroencephalography (EEG), impairment of motor activity, incoordination, hypoglycorrhachia, microencephaly, decreased brain glucose uptake and a decrease by half of brain GLUT1 protein (Wang et al., 2006). In a further analysis, the heterozygous KO mice exhibited postnatal brain weight deceleration, development of reactive astrogliosis and decreased hippocampal volume (Ullner et al., 2009). Another *Glut1*-deficient mouse generated with a gene-trap method exhibited no visible abnormalities, whereas expression levels of *Glut1* and MCTs at the neonatal stage [postnatal day (P)0] were altered compared to that in wild-type mice (Ohtsuki et al., 2006).

N-ethyl-N-nitrosourea (ENU) is an effective chemical mutagen that introduces single-base-pair changes into genomic DNA (Kohler et al., 1991; Provost and Short, 1994). The point mutations that are induced by ENU are able to give rise to a large variety of phenotypes, ranging from a complete or partial loss of function to a gain of function. Several large-scale saturation-ENU mutagenesis projects have been undertaken in order to generate large numbers of mutants that will allow gene functions to be systematically investigated *in vivo* (Funato et al., 2016; Hrabé de Angelis et al., 2000; Nolan et al., 2000). In our ENU mutagenesis program, we identified various mouse mutants that mimic human diseases (Furuse et al., 2010; Masuya et al., 2005, 2007a,b; Inoue et al., 2004; Furuichi et al., 2011; Furuse et al., 2012; Toki et al., 2013; Sato et al., 2010). We conducted behavioral screenings that included the open-field test, passive-avoidance test and home-cage activity test (Wada et al., 2010). In the passive-avoidance test, we isolated a mutant mouse, M100200, which exhibited learning deficiencies.

In the present study, we performed a genetic analysis of the M100200 mouse and identified a missense mutation where serine was substituted with proline at the 324th residue in the GLUT1 protein; we designated the missense mutation as *Glut1^Rgsc200^.* Heterozygotes of the *Glut1^Rgsc200^* mutant showed various phenotypes, including a reduction in body size, deficits in contextual learning, convulsive seizures, immobility during seizures, decreased cerebrospinal fluid (CSF) glucose values and abnormal EEG patterns. Additionally, an *in vivo* kinetic analysis of glucose by using positron emission tomography with 2-deoxy-2-[fluorine-18]fluoro-D-glucose (^18^F-FDG PET) imaging revealed decreased glucose transport, enhanced hexokinase activity and enhanced glucose use in the brain of heterozygotes of the *Glut1^Rgsc200^* mutant. The *Glut1^Rgsc200^* mutant mouse could be a useful tool for elucidating the molecular mechanisms of GLUT1DS.

## RESULTS

### Isolation of a mutant that exhibited learning deficits and seizures in passive-avoidance screening

**Fig. 1.**
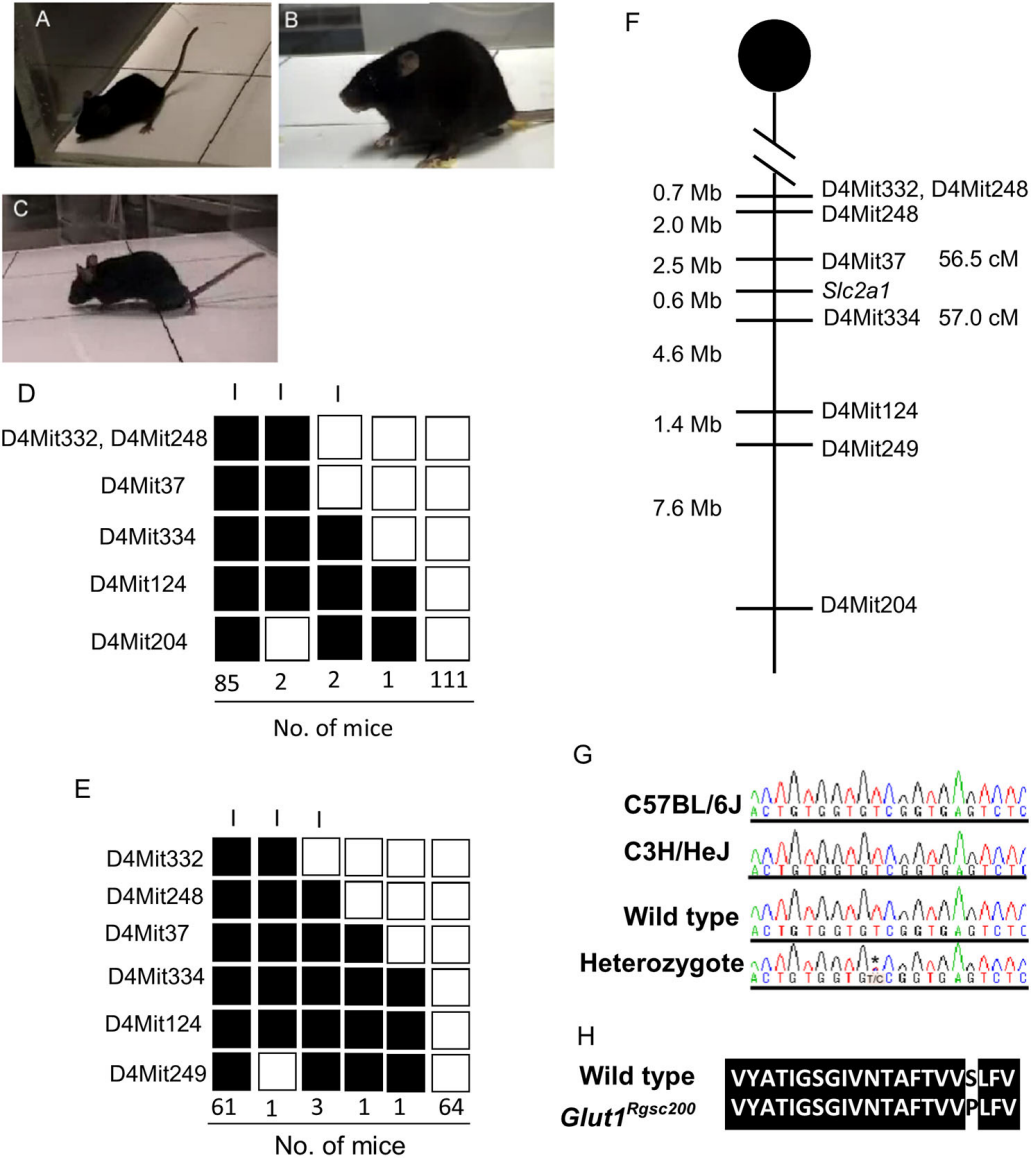
**Mutant mouse line M100200 carries a missense mutation in the *Glut1* gene.** (A-C) Visible seizures observed in the M100200 mutant. Immobility (A) and convulsive seizure (B) started suddenly with vocalization, ataxic gait (C), lowered posture (C) and slow movement. The symptoms started 30-60 min after mice were transferred to a new home cage or open field. When the mutants are immobile, they continuously open their eyes and sometimes move their vibrissae. (D) Haplotype analysis of the N2 progeny of M100200 crossed with B6. Genetic markers are listed on the left side of the panel. The black boxes represent the homozygotes of B6 and the white boxes represent the heterozygotes of B6 and D2. The numbers of progeny that inherited each haplotype are shown at the bottom. ‘I’ indicates mouse groups that exhibited immobility. (E) Haplotype analysis of N4 and N5 progeny of M100200 crossed with C3 mice. Genetic markers are listed on the left side of the panel. The black boxes represent the heterozygotes of B6 and C3, and the white boxes represent the homozygotes of C3. The numbers of progeny that inherited each haplotype are shown at the bottom. ‘I’ indicates the mice groups that exhibited immobility. (F) Genetic map of M100200 constructed from the backcrossed progeny of G1 mice. Genetic markers are listed on the right. (G) Sequence traces from wild types and heterozygotes. The T-to-C substitution is highlighted with an asterisk and corresponds to a point mutation in exon 7 of *Glut1*. (H) Alignment of the 8th transmembrane domain of wild-type GLUT1 protein with that of the mutant allele. Conserved amino acid sequences are highlighted in black. The T-to-C mutation results in an amino acid substitution in the 8th transmembrane domain of GLUT1.

We previously reported detailed results of passive-avoidance screening in the large-scale ENU mutagenesis program conducted at the RIKEN Genome Science Center (GSC) (Wada et al., 2010). In brief, 1998 G1 mice were tested to screen for learning-deficient mice in the passive-avoidance test; 31 phenodeviants were isolated. One of the phenodeviants, M100200, and its progeny exhibited not only learning deficiency but also immobility (Fig. 1A, Movie 1), convulsive seizures (Fig. 1B, Movie 1) and ataxic gait (Fig. 1C, Movie 1). The seizures were induced by transferring the mouse to new environments, such as unfamiliar home cage or open field. The M100200 was backcrossed with C57BL/6J (B6) females and 86 offspring (N2 progeny) were generated. Then, 74 mice of the N2 population were used for the inheritance test. Based on the appearance of immobility, N2 progeny were divided into a normal group or an abnormal group: normal, 40 (female, 19; male, 21) and abnormal, 35 (female, 17; male, 18). This result indicated that the immobility observed in the phenodeviant was inherited by the next generation in an autosomal dominant manner. A total of 201 N2 progenies were generated by backcrossing to B6 and used for linkage analysis. Using the N2 progeny, the causative locus was mapped to a region between D4Mit37 and D4Mit204 (Fig. 1D). Additionally, 132 of N4 to N6 progenies were generated by backcrossing N2 mice to C3H/HeJ (C3) subsequently (Fig. S1B). Using this population, a causative locus was mapped between D4Mit248 and D4Mit249 (Fig. 1E). Finally, by integrating the two linkage maps above, the mutated gene was mapped to a region between D4Mit37 and D4Mit249 (Fig. 1F). A search of the Ensembl mouse genome database revealed that 163 genes were located between D4Mit37 and D4Mit249, and the functions of these genes were investigated from the literature. Based on a list of keywords related to ataxia and seizure, we narrowed the number down to 14 candidate genes (Table 1), and one of them, *Slc2a1* (glucose transporter 1; *Glut1*), was a strong candidate. The *Glut1* gene encodes GLUT1 protein, and sequence analysis of *Glut1* revealed a T-to-C mutation in exon 7 of the gene (Fig. 1G). This mutation resulted in a substitution of serine with proline at residue 324 (Fig. 1H). We designated the *Glut1* mutant allele as *Glut1^Rgsc200^*.

**Table 1.**
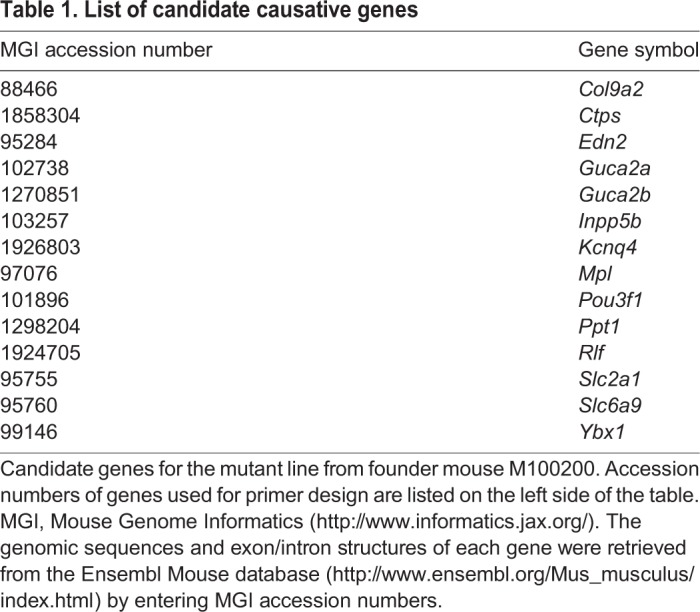
**List of candidate causative genes**

### Transcription of the *Glut1* gene was increased in *Glut^Rgsc200^* heterozygotes

**Fig. 2.**
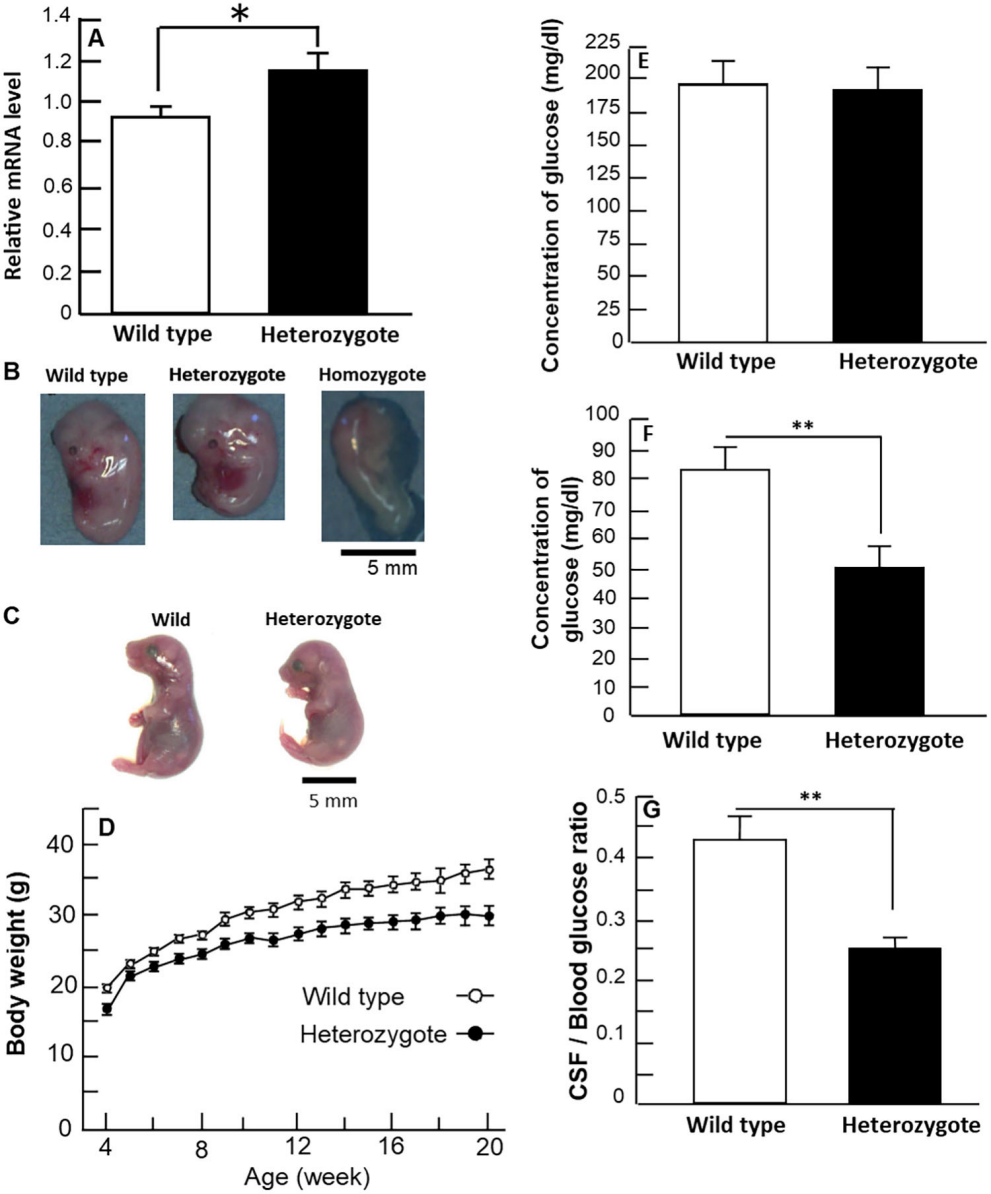
**Embryonic lethality in homozygotes, and body weight loss, increased transcription of the *Glut1* gene and decreased level of CSF glucose in heterozygotes.** (A) Expression of the *Glut1* gene in the forebrain. The average mRNA level of *Glut1* relative to β-actin is shown. The *Glut1* mRNA obtained from forebrain was increased in heterozygotes relative to wild types. Male mice, *n*=4 of each genotype. Student's *t*-test, *t*_6_=−2.908, *P*<0.03. (B) Macroscopic observation of fetuses in uteruses at E13.5. Wild types (left), heterozygotes (middle) and homozygotes (right) conformed to Mendel's law in uteruses of recipient ICR females. Homozygote fetuses exhibited abnormal morphology and developmental defects but the exterior morphology of heterozygote fetuses was normal. (C) Macroscopic observation of fetuses in uteruses at E17.5. No homozygotes existed in uteruses of recipient ICR females. Morphological abnormality was not observed exteriorly in wild-type (left) and heterozygote (right) fetuses. (D) Age-dependent body-weight change of wild types and heterozygotes. Body weight of the wild-type and heterozygote mice were measured after the mice had been weaned (4 weeks of age). Body weight of the heterozygotes was significantly decreased relative to wild types (repeated-measures ANOVA). Significant interaction between the genotype of *Glut1* and age was not detected. Male mice, *n*=10 of each genotype. Repeated measures ANOVA, genotype, *F*_1,306_=158.450, *P*<0.0001; age, *F*_1,16_=39.798, *P*<0.0001; interaction between genotype and age, *F*_1,16_=1.021, *P*>0.4. (E) Blood glucose level. There was no significant difference between wild-type and heterozygote mice in blood glucose value. Student's *t*-test, *t*_22_=0.305, *P*>0.76. (F) CSF glucose level. CSF glucose values were significantly decreased in heterozygotes relative to wild-type mice. Student's *t*-test, *t*_22_=3.126, **P<0.005. (G) CSF:blood ratio of glucose value. CSF:blood ratio of glucose value was significantly decreased in heterozygotes relative to wild-type mice. Student's *t*-test, *t*_22_=3.98, **P<0.0007. (E-G) Male mice at 14 weeks of age, *n*=12 of each genotype. (A-G) Error bars represent the s.e.m.

*Glut1* mRNA and GLUT1 protein levels in the adult forebrain were determined by reverse-transcription quantitative PCR (RT-qPCR) and immunoblotting. Quantification of RT-PCR products revealed that the expression level of *Glut1* mRNA was significantly increased in heterozygotes relative to the wild type (Fig. 2A). Conversely, no significant differences were found between the heterozygous and wild-type mice, with GLUT1 protein expression levels of 45 kDa (Fig. S2A,B) and 55 kDa (Fig. S2A,B), respectively.

### Homozygotes of *Glut1^Rgsc200^* are embryonic lethal

In order to examine embryonic lethality of the *Glut1^Rgsc200^* mutant, we mated heterozygous females and males. At embryonic day (E)13.5, wild-type, heterozygous and homozygous fetuses were present in the uterus; wild types and heterozygotes were macroscopically normal (Fig. 2B), whereas homozygotes exhibited abnormal morphology (Fig. 2B). The population of each gamete conformed to Mendel's law (χ^2^=0.503, Table 2). At E17.5, a late pregnancy period, homozygous embryos did not exist in the uterus, whereas wild-type and heterozygous embryos existed. The population of each gamete departed from the expected values based on Mendel's law (χ^2^=0.0002, Table 2). Wild types and heterozygotes were macroscopically normal at E17.5 (Fig. 2C).

**Table 2.**
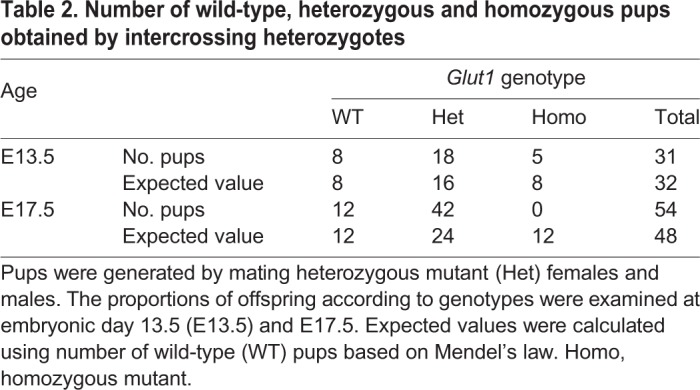
**Number of wild-type, heterozygous and homozygous pups obtained by intercrossing heterozygotes**

### Body weight and CSF-glucose value in heterozygotes of *Glut1^Rgsc200^* mutants were significantly decreased

The average body weight of heterozygotes was significantly decreased relative to wild-type mice (Fig. 2D). Blood glucose values were similar between heterozygotes and wild types (Fig. 2E), although CSF glucose levels were decreased in heterozygotes (Fig. 2F). In addition, the ratio of the CSF glucose value to the blood glucose value was significantly decreased in heterozygotes (Fig. 2G).

### Gross brain histology in *Glut1^Rgsc200^* heterozygotes was normal

No obvious morphological defects were observed in the Nissl-stained tissue sections of the brains of the heterozygotes of *Glut1^Rgsc200^* mutants (Fig. S3).

### Epileptic EEG and abnormal sleep-wake patterns were observed in heterozygotes

**Fig. 3.**
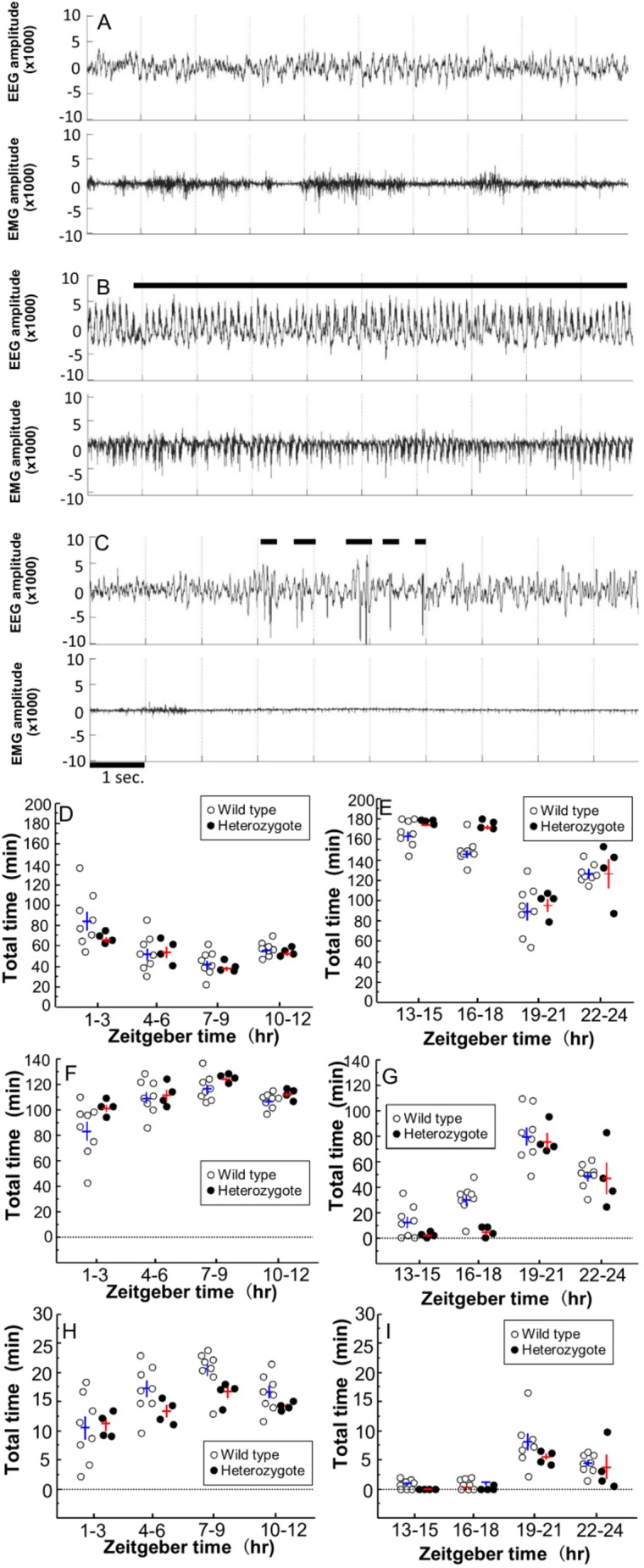
**Electrographic seizures and decreased sleep in heterozygotes of *Glut1^Rgsc200^*.** (A,B) Electrographic seizures observed in heterozygotes. (C) Interictal discharge observed in heterozygotes. (B,C) Horizontal bars indicate appearances of the interictal discharge. (A-C) EEG and EMG recorded from heterozygotes. Upper waveforms show the EEGs and lower waveforms show the EMGs. The EEG signal was amplified 5000-fold and EMG signal was amplified 2000-fold. These amplified signals were converted to the waveform of 16-bit±10 V data. The *x*-axes indicate time scale (in 1-s bins) and *y*-axes indicate amplitude. (D-I) Change of sleep-wake state per 3-h interval. (D,E) Wake, (F-G) NREM sleep, (H,I) REM sleep time. (D-I) Individual data points are plotted. White circles indicate wild types and black circles indicate heterozygotes. The data from individual mice are presented as the group mean±s.e.m. Horizontal lines represent the mean and vertical lines represent the s.e.m. (blue lines, wild type; red lines, heterozygotes). Zeitgeber time (ZT): time lapse from light turned on in the experiment room. Results of the one-way repeated measures ANOVA: (D) wake during light period, effect of genotype, *F*_1,10_=1.5341, *P*>0.25; effect of increment of ZT, *F*_3,30_=10.6735, *P*<0.0001; interaction between genotype and increment of ZT, *F*_3,30_=0.9562, *P*>0.4; (E) wake during dark period, effect of genotype, *F*_1,10_=7.4648, *P*<0.022; effect of increment of ZT, *F*_3,30_=47.9121, *P*<0.0001; interaction between genotype and increment of ZT, *F*_3,30_=1.0681, *P*<0.37737; (F) NREM sleep during light period, effect of genotype, *F*_1,10_=4.9636, *P*>0.05; effect of increment of ZT, *F*_3,30_=9.6638, *P*<0.0001; interaction between genotype and increment of ZT, *F*_3,30_=0.8496, *P*>0.4; (G) NREM sleep during dark period, effect of genotype, *F*_1,10_=7.3703, *P*<0.03; effect of increment of ZT, *F*_3,30_=49.3272, *P*<0.0001; interaction between genotype and increment of ZT, *F*_3,30_=1.4120, *P*>0.25; (H) REM sleep during light period, effect of genotype, *F*_1,10_=2.1955, *P*>0.16; effect of increment of ZT, *F*_3,30_=12.2923, *P*<0.0001; interaction between genotype and increment of ZT, *F*_3,30_=1.3697, *P*<0.2709; (I) REM sleep during dark period, effect of genotype, *F*_1,10_=2.0277, *P*>0.18; effect of increment of ZT, *F*_3,30_=21.7062, *P*<0.0001; interaction between genotype and increment of ZT, *F*_3,30_=0.4969, *P*>0.68.

We recorded EEG in 8 wild-type mice and 4 heterozygotes. In the EEG recording period of heterozygotes, abnormal wave patterns, epileptic waveforms (Fig. 3A,B) and interictal discharges were observed (Fig. 2C), while wild-type mice did not exhibit epileptic EEG patterns. These EEG abnormalities were observed with no obvious epileptic seizures and immobility in the heterozygotes. The heterozygotes spent more time awake and less time in NREM sleep compared to the wild types (Table 3, Fig. 3D-F).

**Table 3.**
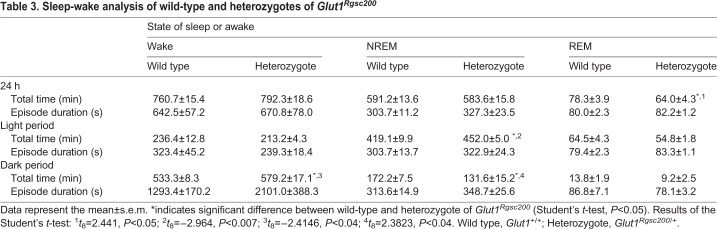
**Sleep-wake analysis of wild-type and heterozygotes of *Glut1^Rgsc200^***

### [^18^F]FDG PET

**Fig. 4.**
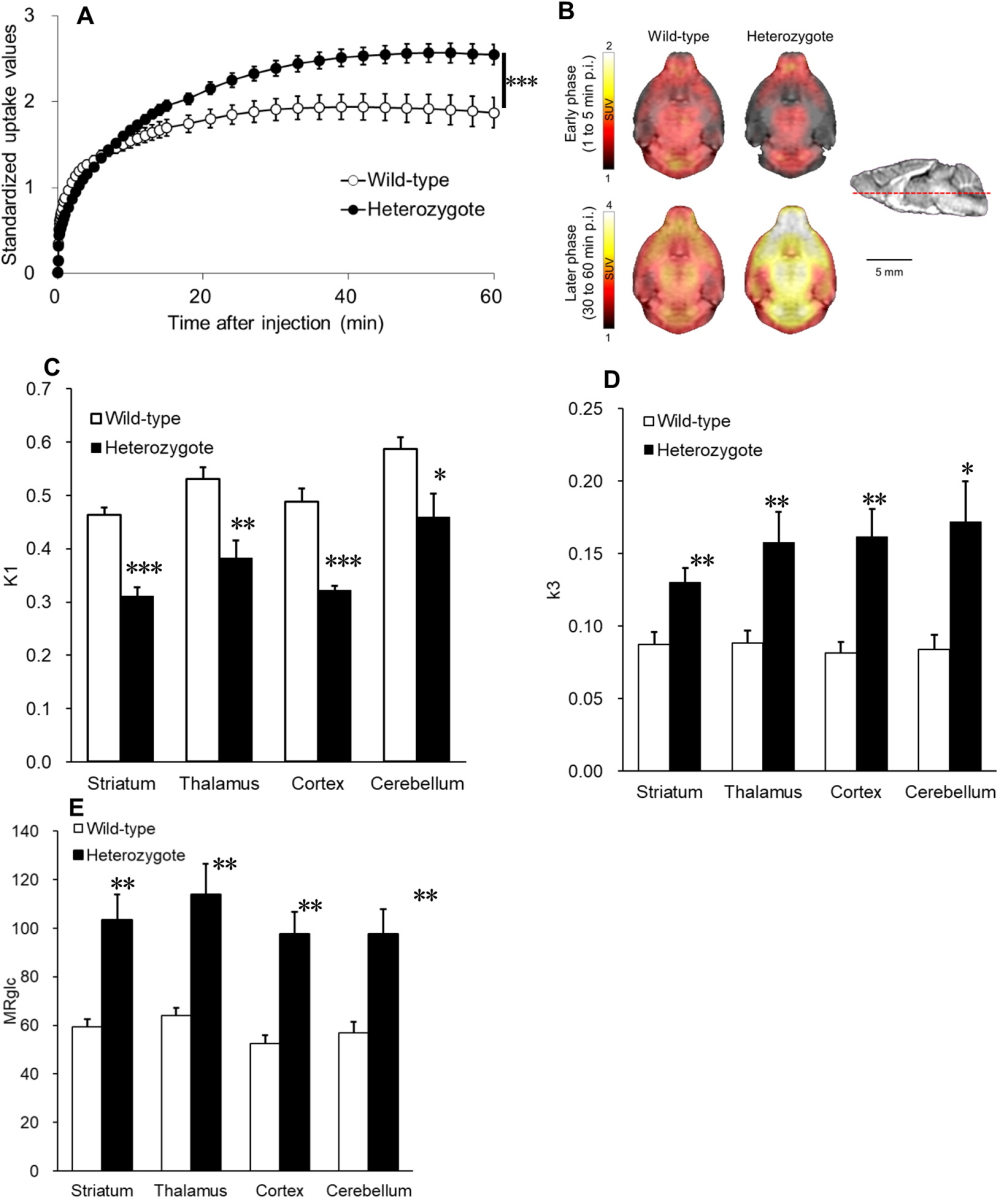
***In vivo* PET imaging with [^18^F]FDG in awake mice.** (A) Mean time-radioactivity curves of [^18^F]FDG in the whole brain. Standardized uptake value (SUV; g/ml) indicates the regional radioactivity (Bq/ml) per injected radioactivity (Bq/g). Genotype, *F*_1,450_=3.862, ***P<0.001, two-way repeated-measures ANOVA. (B) Summed PET images from 1 to 5 min (early phase) and 30 to 60 min (later phase) after injection of [^18^F]FDG were generated by averaging in each group. (C) The *k*_1_ values calculated from time-course change of SUV in various brain regions. (D) The *k*_3_ values calculated from time-course change of SUV in various brain regions. (E) Regional cerebral glucose metabolic rate. (A,C-E) Error bars represent the s.e.m. Male mice, *n*=6; measurements were carried out twice per mouse and mean values were used for statistical analyses; *P<0.05, **P<0.01, ***P<0.001, compared with wild type.

In the heterozygote mice, [^18^F]FDG was slightly taken up into the brain until 5 min after injection at the early phase, but the high accumulation of [^18^F]FDG occurred 30 min post-injection (Fig. 4A,B) [for genotype, *F*_1,450_=3.862, *P*<0.001, two-way repeated-measures analysis of variance (ANOVA)]. To quantitatively assess the kinetics of glucose utilization, we determined the rate constants *k*_1_, *k*_2_, *k*_3_ and *k*_4_, defined, respectively, as the influx rate of glucose into the brain, efflux rate out of the brain, intracellular phosphorylation and intracellular dephosphorylation. We also assessed regional cerebral glucose metabolic rate (rCMRglc) as a proxy of glucose consumption calculated from these rate constants. This kinetic analysis revealed that the *k*_1_ value was significantly reduced in the heterozygotes as compared with wild type, whereas the *k*_3_ value in the heterozygote mice was significantly higher than that in the wild-type mice (Fig. 4C,D). In addition, rCMRglc in the heterozygotes was higher than that in wild-type mice (Fig. 4E).

### Contextual learning was affected in heterozygotes

**Fig. 5.**
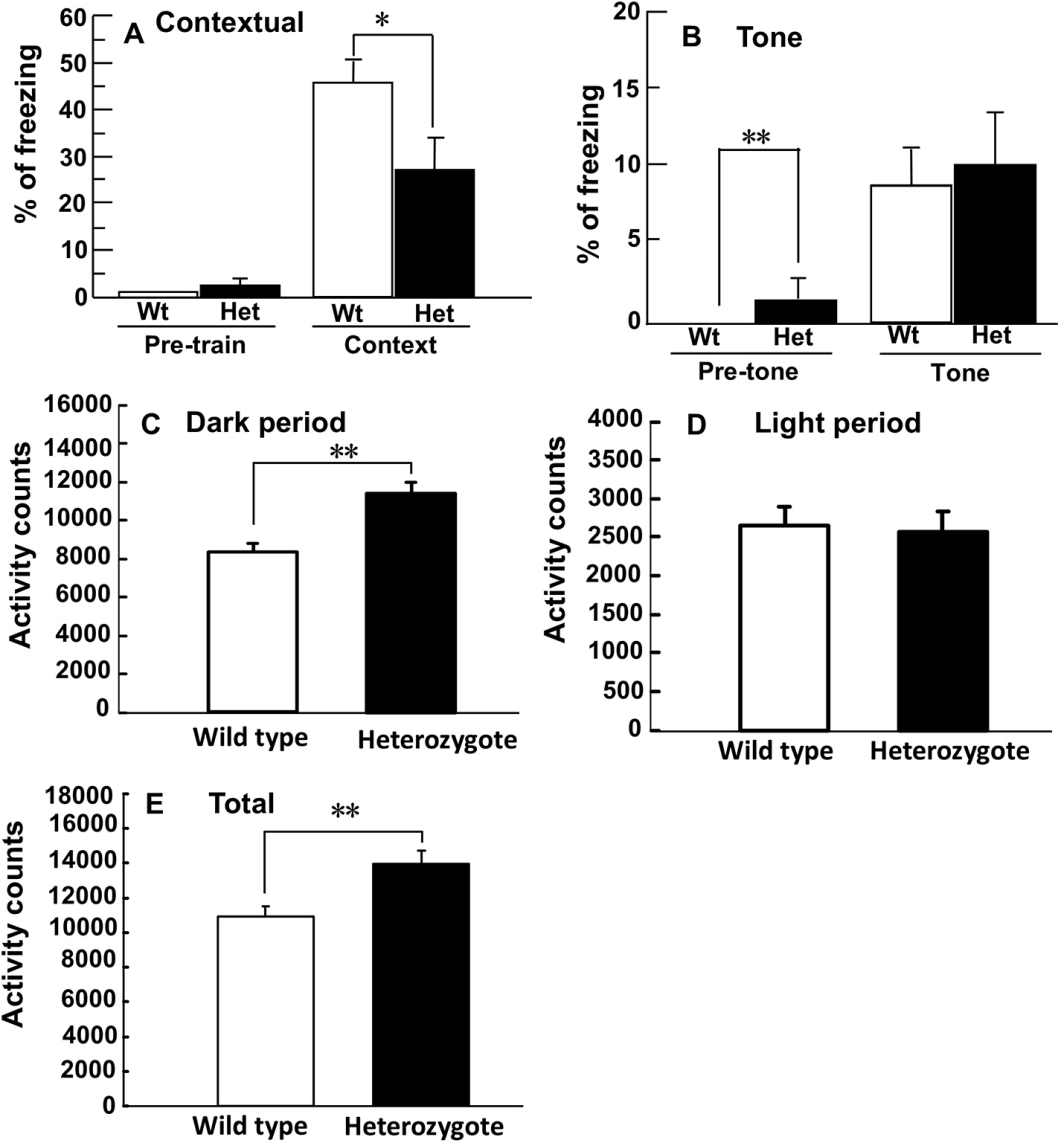
**Behavioral alterations in heterozygotes of *Glut1^Rgsc200^* mutants.** (A,B) Impaired contextual learning and normal tone-dependent learning in heterozygotes. (A) Freezing responses pre- and post-training with 3 tone-shock pairs in box A. Pre-train indicates the freezing levels in box A (the shocking chamber) before the onset of training. Context indicates freezing level after training. Freezing level after training in heterozygotes (Het) was significantly lower than wild-type (Wt) mice. Student's *t*-test, pre-train, *t*_18_=0.809, *P*>0.4; contextual, *t*_18_=2.871, *P<0.02. (B) Freezing responses to tone presentation in box B after 3 tone-shock pairs. Pre-tone indicates the freezing levels in box B after training and before the tone testing. Tone indicates the freezing levels in box B during the presentation of tone. Freezing level in heterozygotes at the pre-tone period was significantly higher than in wild-type mice. Student's *t*-test, pre-tone, *t*_18_=−2.324, *P<0.04; tone, *t*_18_=−0.1739, *P*>0.1. (A,B) Male mice at 11 weeks of age, wild type, *n*=12; heterozygote, *n*=8. (C-E) Locomotor activity of wild types and heterozygotes of *Glut1^Rgsc200^* mice in their home cages. (C) Mean locomotor activity in the home cage during the dark period. Student's *t*-test, *t*_18_=−3.132, **P<0.006. (D) Mean locomotor activity in the home cage during the light period. Student's *t*-test, *t*_18_=0.221, *P*>0.8. (E) Mean locomotor activity in the home cage during the dark and the light period. Student's *t*-test, *t*_18_=−0.39, **P<0.0008. (C-E) Male mice, *n*=10 of each genotype at 10- to 11-weeks of age. (A-E) Error bars represent the s.e.m.

On day 2 of the fear-conditioning test, the heterozygotes displayed a decreased level of freezing relative to wild types in the conditioning chamber (Fig. 5A). On day 3, heterozygote and wild-type mice displayed similar levels of freezing with the tone (Fig. 5B). The results indicate that the *Glut1^Rgsc200^* mutant mice are impaired only in contextual fear conditioning.

### Heterozygotes exhibited an enhancement of spontaneous locomotor activity in their home cage

Heterozygotes exhibited an increased locomotor activity in the home cage (Fig. 5C,E). This increase of locomotor activity was attributable to an increased locomotor activity in the dark phase (Fig. 5C-E).

## DISCUSSION

In the present study, we performed a genetic analysis for the ENU-induced mutant mouse M100200 and identified a missense mutation at the 324th residue of the GLUT1 protein that we designated as *Glut1^Rgsc200^*. Deficient functions of the GLUT1 protein cause infantile epilepsy, which is designated as GLUT1DS. Therefore, the *Glut1^Rgsc200^* mutant mice are expected to be a new model for GLUT1DS, and findings obtained from this mutant strain will help us identify the pathogenic mechanism of GLUT1DS.

The missense mutation S324P in *Glut1^Rgsc200^* is located in the 8th transmembrane domain of the GLUT1 protein (Pascual et al., 2004). Serine is a polar amino acid and proline is a nonpolar amino acid. In addition, expression levels of GLUT1 protein were not changed between heterozygotes and wild types. However, transcription of the *Glut1* gene was increased in the heterozygotes relative to the wild types. Therefore, a drastic change of the polar character of the amino acid residue in the mutated *Glut1* gene may have caused a change in conformation or stability of the amino acid. In fact, decreased levels of CSF glucose and ^18^F-FDG intake were observed in the brain of heterozygous *Glut1^Rgsc200^* mutants. In addition, at least three causative mutations for GLUT1DS were identified in the 8th transmembrane domain of the GLUT1 protein (Pascual et al., 2004).

In ENU mutagenesis, point mutations are randomly introduced into the genome. Therefore, we backcrossed the phenodeviant mice to wild-type mice at least 9 times, carrying out genotype-driven selection at each generation. Backcrossed progenies were used for further phenotyping assays. As a result of the backcrossing, 99.8% of the genetic background of the *Glut1^Rgsc200^* mutant was substituted in un-mutagenized C57BL/6J strains and the N10 mice displayed the previously described phenotypes. Therefore, the S324P amino acid substitution is expected to contribute strongly to the GLUT1DS-like phenotypes, such as visible seizures and learning deficits, observed in the *Glut1^Rgsc200^* phenodeviant mice. However, we cannot exclude the possibility that the *Glut1^Rgsc200^* phenotypes may also have been induced by mutations in genes neighboring *Glut1*.

In the present study, we found the following unique phenotypes of the *Glut1^Rgsc200^* mutants: a significant reduction in body weight, spontaneous visible seizures, deficits in contextual learning, an increased locomotor activity in the home cage, a shorter duration of rapid eye movement (REM) and NREM sleep, longer intervals of the wake period, and abnormal glucose kinetics in the brain. Conversely, a decrease of CSF glucose levels, epileptic EEG waveforms, interictal EEG discharges, and embryonic lethality were common among *Glut^Rgsc200^* and targeted mutants of *Glut1* (Wang et al., 2006). The shortened period of NREM and REM sleep and prolonged duration of the wake period may resemble the sleep disturbances commonly observed in patients with GLUT1DS (De Giorgis and Veggiotti, 2013) and other epilepsy disorders (Schmitt, 2015).

Ketone bodies cross the BBB and are consumed under metabolic ketosis as an alternative energy source to glucose in the brain. Therefore, seizures in GLUT1DS patients are suppressed by treatment with a ketogenic diet (Klepper et al., 2004). In the present study, we did not examine the effects of fasting-induced ketosis on body weight or the glycemic indices of blood and CSF. Phenotypic analyses of *Glut1^Rgsc200^* mutants under metabolic ketosis would reinforce the value of the mutant strain as a GLUT1DS model.

Under anesthetized conditions, the metabolic rate of glucose in the brain was markedly suppressed relative to that in conscious conditions (Mizuma et al., 2010), although it was difficult to accurately evaluate the glucose kinetics under that condition. Therefore, we employed a method for PET imaging under non-anesthetized conditions (Mizuma et al., 2010) in the present study and observed a suppressed *k*_1_ value (an indicator of glucose intake) in the brain of the heterozygotes compared with wild-type mice. On the other hand, the *k*_3_ value (an indicator of the hexokinase activity) in the heterozygote mice was significantly higher than that in the wild-type mice. In addition, rCMRglc (an index of glucose use in the brain) was increased in the brain of heterozygotes. Despite the alterations of the glucose kinetics described above, there was no difference in the GLUT1 protein level in the brain between the mutant and wild-type mice. The reduction in glucose transportation seems to induce a decrease in glucose consumption, which is required for neural activation; however, glucose use was enhanced in the mutant mice. These results suggest that the increased glucose metabolism resulted from the compensatory action of glucose transporter dysfunction to maintain neuronal activity and metabolism intact. Previous PET studies of a GLUT1DS animal model have reported that gene-targeted mutant *Glut1* heterozygous mice showed globally low [^18^F]FDG uptake levels in the brain (Wang et al., 2006). Similarly, [^18^F]FDG-PET studies in patients with GLUT1DS have reported a widespread decrease in [^18^F]FDG uptake throughout the entire brain. Notably, glucose hypometabolism was observed in thalamic, cerebellar and neocortical regions (Pascual et al., 2004). In addition, a comparative study of [^18^F]FDG-PET imaging between GLUT1DS and healthy subjects revealed regional glucose hypometabolism in the thalamus, cerebellum, temporal cortex and central lobule of GLUT1DS patients (Akman et al., 2015). By contrast, our GLUTDS1 model mice showed high glucose utilization in the brain. This discrepancy between the results of previous experiments and our own PET imaging experiments is thought to be due to the different genic locations of the *Glut1* mutations inducing different results in GLUT1 protein expression level and/or functionality. In the present study, we demonstrated that *Glut1* mutant mice have a dysfunctional glucose transporter with abnormal glucose metabolism in the brain under conscious conditions. Additionally, positional information of neuronal activity in the brain of wild-type mice and heterozygous *Glut1* mutants will indicate the pathological region in the brain of GLUT1DS patients. Therefore, the PET imaging enables real-time monitoring of glucose kinetics in genetically manipulated mice and will provide new diagnostic insights into GLUT1DS. *Glut1^Rgsc200^* mutants mimicked the major symptoms of GLUT1DS described above similarly to known *Glut1* mutants that have been previously reported. Patients of GLUT1DS carry various types of mutations in the *GLUT1* gene, including missense, nonsense and deletions (Pascual et al., 2004); this indicates that the pathogenesis and symptoms of GLUT1DS are diverse (Pearson et al., 2013). Therefore, various animal models of GLUT1DS should be generated. The missense mutation T310I, which falls close to the *Glut1^Rgsc200^* mutation and affects the protein's 8th transmembrane domain, is known to cause infantile seizures, decreased CSF glucose levels and a learning disability (Akman et al., 2015). The *Glut1^Rgsc200^* mutant may be a useful tool for elucidating the molecular mechanisms of GLUT1DS and may help develop therapeutic drugs for GLUT1DS.

## MATERIALS AND METHODS

### Ethics statement of animal experiments

All procedures described here were reviewed and approved by the Institutional Animal Care and Use Committee of RIKEN Tsukuba Branch and were performed in accordance with the RIKEN Guiding Principles for the Care and Use of Laboratory Animals (No. 10-013).

### ENU mutagenesis and animal production

Large-scale mouse ENU mutagenesis was conducted as described previously (Furuse et al., 2010, 2012; Masuya et al., 2005) with some modifications. C57BL/6J (B6), DBA/2J (D2) and C3H/HeJ (C3) mice were purchased from a commercial supplier (CLEA Japan, Inc., Tokyo, Japan). The following strategy is outlined in Fig. S1. B6 males were treated with ENU (150-250 mg/kg body weight) by intraperitoneal (i.p.) injection and crossed with D2 females. The progeny generated by this cross were designated as G1 mice (Fig. S1A). We collected baseline data from the G1 mice and performed phenotypic screening on the G1 mice as previously described (Wada et al., 2010). Outliers isolated in the phenotypic screening were designated as phenodeviants. The phenodeviants were backcrossed to B6 mice for inheritance testing and gene mapping. Progenies generated from phenodeviants and B6 mice were designated as N2 (Fig. S1A). The N2 mice that exhibited phenotypes observed in the phenodeviants were then backcrossed to C3 mice for linkage analysis (Fig. S1B). In addition, the N2 mice were backcrossed to B6 at least 9 times to unify the genetic background and eliminate ENU-induced mutations that did not link to the phenodeviants’ abnormal phenotypes (Fig. S1C).

### Behavioral screening of the founder mouse in ENU mutagenesis program

A detailed procedure of the behavioral screenings and criteria used to detect outliers from the G1 population in the RIKEN-ENU mutagenesis program was described previously (Wada et al., 2010). Briefly, we performed three behavioral tests in the following order: at 8 weeks of age, a home-cage activity test for screening spontaneous activity and rest/activity cycles; at 9 weeks of age, an open-field test for the screening of novelty-induced activity that reflects fear, anxiety and exploration; at 11 weeks of age, a passive-avoidance test for screening shock avoidance response that reflects learning and memory.

In the passive-avoidance test, the apparatus consisted of a light chamber and a dark chamber (PA-3002AD, O'Hara & Co. Ltd, Tokyo, Japan). Its floor was composed of stainless steel bars connected to an electric-shock generator (SGA-2050, O'Hara & Co. Ltd). The mouse was placed in the light chamber, and the guillotine door was opened 20 s later. During conditioning, an electric shock (0.15 or 0.3 mA) was delivered across the floor immediately after the mouse entered the dark chamber. The shock delivery was terminated when the mouse escaped into the light chamber. Mice that did not enter the dark chamber within 180 s during conditioning were eliminated from the analysis. The latency of entering the dark chamber was automatically recorded by a photocell beam-break detector (O'Hara & Co. Ltd). On the following day, a retention test was conducted. If the mouse entered the dark chamber within 300 s during the retention test session, an electric shock was delivered, and this test was repeated 1 week later. If the mouse did not enter the dark chamber for 300 s (the learning criterion), the retention test was terminated. We performed the retention test once a day, for a maximum of 3 days, until the mouse met the learning criterion.

### Genetic mapping

In order to determine the mutation, we phenotyped the N2 mice for immobility or convulsive seizure observed while the mice were transferred to a new environment. Single nucleotide polymorphism (SNP) markers spaced at 10 cM were chosen from the Mouse SNP database (http://www.broad.mit.edu/snp/mouse/) and used for genome-wide scanning: rs3022775, rs3090110, rs3022803, rs3022831, rs3022839, rs3089480, rs3022886, rs3022886, rs3022901, rs3022937, rs3022946, rs3022955, rs3088635, rs3023238, rs3090147, rs3090379, rs3022972, rs3022977, rs3089558, rs3022988, rs3022997, rs3023010, rs3023026, rs3023044, rs3090396, rs3090397, rs3090525, rs3023051, rs3023057, rs3090572, rs3023071, rs3023084, rs3023094, rs3023105, rs3023129, rs3023148, rs3023157, rs3090007, rs3023185, rs3023187, rs3023188, rs3023202, rs3091100, rs3089531, rs3089156, rs3023233, rs3023242, rs3088857, rs3023250, rs3089172, rs3023256, rs3023265, rs3023278, rs3090222, rs3023316, rs3089465, rs3023346, rs3023377, rs3023379, rs3091203, rs3023380, rs3089498, rs3090371, rs3090029, rs3090029, rs3090999, rs3089783, rs3023410, rs3090790, rs3023414, rs3091174, rs3023418, rs3023421, rs3088710, rs3090882, rs3089786, rs3089786, rs3023110, rs3023444, rs3090283, rs3023460, rs3090636, rs3023467, rs3023477, rs3090751.

We used microsatellite and SNP markers for a detailed linkage analysis. We determined the genomic structures of the candidates with the mouse Ensembl database (http://www.ensembl.org/Mus_musculus/index.html) and performed direct sequencing of coding regions of the candidate genes.

### Genotyping of mutated *Glut1*

The T-to-C point mutation in exon 7 of *Glut1* was genotyped by using the allele-specific primer polymerase chain reaction (ASP-PCR) (Petkov et al., 2004) and direct sequencing. ASP-PCR was performed with the following primers: forward primer for wild-type and mutant alleles, 5′-CCTGCCTGGGAAGTGTTGAT-3′; reverse primer for the wild-type allele, 5′-ACCAAAAGCGAGACTCGA-3′; reverse primer for the mutant-type allele, 5′-ACCAAAAGCGAGACTCACCTG-3′.

### Quantitative PCR of *Glut1*

Mice were killed by cervical-vertebra dislocation and the forebrain was collected. The forebrain was quickly immersed in liquid nitrogen and kept at −80°C. Brain tissue (50-100 mg) was homogenized in TRIZOL (Invitrogen, Carlsbad, CA, USA) using Multi-Bead shocker (Yasuikikai, Osaka, Japan). Chloroform (0.2 ml) was added into homogenate. The tube containing homogenate was shaken by hand vigorously for 15 s and centrifuged for 10 min at 10,000 rpm (9100 ***g***) at 5°C. The aqueous phase was transferred into a fresh tube and 0.5 ml of isopropyl alcohol was added into the tube. The tube was shaken gently and centrifuged at 10,000 rpm for 10 min at 4°C. Isopropyl alcohol was removed and 1 ml of 70% ethanol as was added to the aqueous fraction, and the tube was shaken gently. The tube was centrifuged at 5000 rpm for 5 min at 4°C and supernatant removed. The pellet was dried and resolved into water and stored at −80°C. Total RNA was isolated from the brain of wild-type and heterozygote mice at 30 weeks of age as described above. The RT-qPCR analysis was conducted by Dragon-genomics center (Takara Bio Inc., Shiga, Japan). In brief, 600 ng of total RNA was used for reverse transcription and cDNA was amplified by PCR reaction. In each reaction, a SYBR RT-PCR Kit (perfect real time) (Takara Bio Inc.) was used. The PCR reaction was performed by Smart Cycler version II (Takara Bio Inc.) with the following primers: forward primer for *Glut1*, 5′-CTTCATTGTGGGCATGTGCTTC-3′, reverse primer for *Glut1*, 5′-AGGTTCGGCCTTTGGTCTCAG-3′, forward primer for β-actin, 5′-TGACAGGATGCAGAAGGAGA-3′, reverse primer for β-actin, 5′-GCTGGAAGGTGGACAGTGAG-3′. The PCR product was stained by SYBR Green I (Schneeberger et al., 1995) and detected in real time. The expression level of *Glut1* was normalized by β-actin mRNA level.

### Immunoblotting of GLUT1 protein

We prepared brain tissue samples as reported previously (Badr et al., 1999), with slight modifications. Briefly, mice were sacrificed by cervical vertebra dislocation and the forebrain was collected. Forebrain (20 mg) was homogenized 10 strokes using a glass-Teflon homogenizer at a 1:15 dilution weight per volume in a solution containing 0.25 M sucrose, 10 mM Tris, 0.5 mM EGTA, pH 7.4. Homogenates were centrifuged at 2000 ***g*** for 5 min and the resulting post-nuclear supernatants were used in western blot analysis. Fifty micrograms of protein were loaded per lane in blots. The blotted membranes were blocked with EzBlock (ATTO Corporation, Tokyo, Japan) for 1 h and washed with Tris-buffered saline (TBS; pH 7.5) containing 0.1% Tween 20 (TBS-T) 3 times. The membrane was probed with rabbit anti-Glut-1 antibody (1:500; catalog number, AB1340; lot number, 23071304; Millipore, MA, USA) or rabbit anti-β-actin antibody (1:5000; catalog number, 622102; lot number, B106202; clone number, Poly6221; Sigma-Aldrich, Inc., MO, USA). After washing 3 times with TBS-T, the membrane was incubated with alkaline-phosphatase-conjugated goat anti-rabbit IgG (1:5000; catalog number, 12-448; lot number, 30190; Upstate Biotechnology, NY, USA) and signals were detected with NBT/BCIP substrates.

### Generation of *Glut1^Rgsc200^* heterozygotes

Phenodeviant mice were backcrossed to C57BL/6JJcl mice (CLEA Japan, Inc.) at least 9 times. At each generation, the *Glut1^Rg^^sc^^200^* allele was genotyped and the heterozygous mutants were mated with C57BL/6JJcl mice. The backcrossed progenies were used for detailed phenotyping assays (Fig. S1C).

### Generation of *Glut1^Rgsc200^* homozygotes

In order to generate *Glut1^Rgsc200^* homozygotes, we conducted *in vitro* fertilization (IVF) and embryo transfer (ET) as described previously (Furuse et al., 2017). Briefly, adult males of heterozygotes were used as a source of sperm, and 8-week heterozygote females were used as a source for oocytes for IVF. ICR (CLEA Japan, Inc.) females were used as pseudopregnant recipients for ET. At E13.5 and E17.5, embryos were isolated by caesarean section and macroscopically examined.

### Brain gross histology

We performed histological analysis of the brains as described previously (Furuse et al., 2010). Mice were deeply anesthetized with i.p*.* pentobarbital injection and then fixed by transcardial perfusion with a solution of 4% paraformaldehyde and 0.5% picric acid in PBS. The brain was removed and coronal or parasagittal sections (70 μm thick) were prepared with a vibratome. Sections were stained with NeuroTrace 500/525 (fluorescent Nissl stain; Invitrogen, Carlsbad, CA, USA) according to the manufacturer's instructions. Fluorescence images were obtained with a confocal laser-scanning microscope (BX62) (Olympus, Tokyo, Japan).

### Blood glucose and CSF glucose measurement

Mice were deeply anesthetized with a mixture of ketamine (Daiichi Sankyo Co., Ltd, Tokyo, Japan) and xylazine (Bayer Health Care, Leverkusen, Germany). After the anesthesia, we collected 20 μl mouse blood via the tail tip and measured glucose levels by using GLUCOCARD alpha (ARKRAY Inc., Kyoto, Japan). CSF was isolated from the cisterna magna as described previously (Boje, 2001), with a slight modification. Briefly, after the deep anesthesia described above, we cut the skin carefully over the back of the skull and outstretched the neck of the mouse*.* Neck muscles were removed to expose the white atlanto-occipital membrane that covers the cisterna magna. Ten microliters of CSF were collected from the cisterna magna with a 26-G hypodermic needle that was connected to a micropipette via a polyethylene tube, and, as immediately as possible, the glucose concentration in the CSF was measured by using GLUCOCARD alpha. A CSF:blood ratio of the glucose value was calculated as follows: CSF:blood ratio=Concentration of CSF glucose/Concentration of blood glucose.

### EEG/EMG recording

#### Surgery and EEG/EMG recording

Male heterozygotes (*n*=4, 14 weeks old, 24-30 g at the time of surgery) and wild-type C57BL/6J (*n*=8) were anesthetized with isoflurane (4% for induction, 2% for maintenance). After the cranium was exposed, 4 electrode pins were lowered to the dura mater under stereotaxic control, and 2 flexible wires for electromyography (EMG) recording were inserted in the neck muscles and then attached to the skull with dental cement. The electrodes for the EEG signals were positioned over the frontal and occipital cortices (frontal: 0.5 mm anterior to bregma, 1.27 mm lateral to midline; occipital: 0.5 mm anterior to lambda, 1.27 mm lateral to midline). The mice were kept on a heating pad until fully recovered, and then housed individually. Buprenorphine (0.03 mg/kg body weight) was given subcutaneously to reduce pain. Seven days after surgery, the mice were tethered to a counterbalanced arm (Instech Laboratories, Plymouth Meeting, PA, USA) that allowed for free movement and exerted minimal weight. All mice were allowed 14 days of recovery from the surgery and habituation to the recording conditions. Food was available *ad libitum* except during the phase of the fasting test. Agar gel (Napa Nectar^TM^, System Engineering Lab Group, CA, USA) was used for water supply. The recording room was kept under a 12 h light-dark cycle and a constant temperature (24-25°C). To examine sleep-wake behavior under baseline conditions, EEG/EMG signals were recorded for 2 consecutive 24-h periods from the onset of the light phase.

#### Sleep-wake analysis

The signals of EEG and EMG were amplified and filtered (EEG: 0.5-100 Hz; EMG: 5-300 Hz) using an amplifier (AB-611J, Nihon Kohden, Japan), then digitized at a sampling rate of 250 Hz. The recording was controlled under a custom software written in LabView (National Instruments, Austin, TX, USA). The state in each 20-s epoch was classified as wakefulness, NREM sleep or REM sleep using a custom software script written in MATLAB (MathWorks, Natick, MA, USA) and visual inspection, according to the standard criteria of rodent sleep (Radulovacki et al., 1984). The total time spent in wakefulness, NREM and REM sleep was derived by summing the total number of 20-s epochs in each state. Mean episode durations were determined by dividing the total time spent in each state by the number of episodes of that state.

### ^18^F-FDG PET imaging

To investigate the kinetics of glucose in the brain, PET imaging with [^18^F]2-fluoro-2-deoxy-D-glucose (FDG), an analog of glucose, was performed according to the method in our previous report (Mizuma et al., 2010). Briefly, after attachment of the head holder, which was surgically attached under 1.5% isoflurane anesthesia, the mouse recovered from the surgery in their home cage for at least 1 week prior to PET scanning. During the PET imaging experiment, a polyethylene catheter with 31-G needle was inserted into their tail vein, and the blood glucose level was measured prior to the PET scanning. PET imaging was performed using a PET scanner for small animals (microPET Focus-220, Siemens Medical Systems, TN, USA). A transmission scan was performed for 30 min using a ^68^Ge–^68^Ga pin source (18.5 MBq; Siemens Medical Systems) for attenuation correction. [^18^F]FDG (0.7-1.0 MBq/g body weight) was intravenously injected automatically via the catheter for 10 s using a syringe pump (PHD-2000; Harvard Apparatus Inc., MA, USA). Emission data were then acquired for 60 min using a 3D list-mode method of data acquisition and were subsequently sorted into 45 frames (10×3 s, 3×10 s, 6×30 s, 11×60 s and 15×180 s). The emission data were reconstructed using a filtered back-projection (FBP) algorithm with attenuation correction, no scatter correction, and smoothing by a Gaussian kernel with 3 mm full width at half maximum in all directions. Cerebral [^18^F]FDG kinetics were calculated by 3-compartmental modeling using the ^18^F radioactivities in the cardiac ventricle as an input function derived from dynamic PET images and calculated rCMRglc (μmol/min/100 g) and rate constants of [^18^F]FDG *k*_1_-*k*_4_: *k*_1_ (ml/min/g) and *k*_2_ (min^–1^) values indicate intra- and extracellular transportation mediated by glucose transporter across cytomembranes, respectively; *k*_3_ (min^–1^) and *k*_4_ (min^–1^) represent which [^18^F]FDG was phosphorylated by hexokinase and diphosphorylated by glucose-6-phosphatase, respectively.

### Behavioral tests

#### Fear-conditioning test

We performed a fear-conditioning test as previously described (Furuse et al., 2017) with slight modification. On the training day (day 1), each mouse was placed in a shock chamber with white walls (O'Hara & Co. Ltd) (box A) and, after 120 s, 4 tone-shock pairs were given at 90 s intervals. Each tone-shock pair consisted of a tone (70 dB, 10 kHz) for 30 s and a foot shock for 2 s at 0.5 mA. The foot shock was presented to mice during the last 2 s of the tone. On day 2, each mouse was placed back in box A for 6 min to measure contextual freezing. On day 3, each mouse was placed in a white transparent chamber (box B) and, 180 s later, 180 s tones were delivered. Freezing was measured during the first 180 s in box B, representing the response to an unconditioned context, and then during the next 180 s in the presence of the conditioned tone.

#### Home-cage activity test

A home-cage activity test was performed as described previously (Furuse et al., 2012). Briefly, each mouse was placed alone in a testing cage (227×329×133 mm) under a 12-h light-dark cycle (light on at 8:00 a.m.) and had free access to both food and water. After 1 day of acclimation, spontaneous activity in the cage was measured for 5 days (starting at 08:00) with an infrared sensor (AB system 4.0, Neuroscience Co., Ltd, Tokyo, Japan).

### Statistical analyses

For the statistical analyses of the results of EEG recordings, a Student's *t*-test and repeated measures ANOVA were performed and differences were considered to be statistically significant at *P*-values <0.05. In the behavioral analyses, 2-way (genotype and experimental factor: between subjects) or 3-way (genotype, sex and experimental factor: between or within subjects) ANOVA with Fisher's PLSD post-hoc analysis and Student's *t*-test were used for statistical analyses. Differences were considered to be statistically significant at *P*-values <0.05.

## Supplementary Material

Supplementary information
