# Supplementary material for: A new mouse model of GLUT1 deficiency syndrome exhibits abnormal sleep-wake patterns and alterations of glucose kinetics in the brain
Source: Dis Model Mech. 2019 Sep 12;12(9):dmm038828. doi: 10.1242/dmm.038828 (PMC6765196; doi:10.1242/dmm.038828)
Supplement: Supplementary information [file dmm-12-038828-s1.pdf]

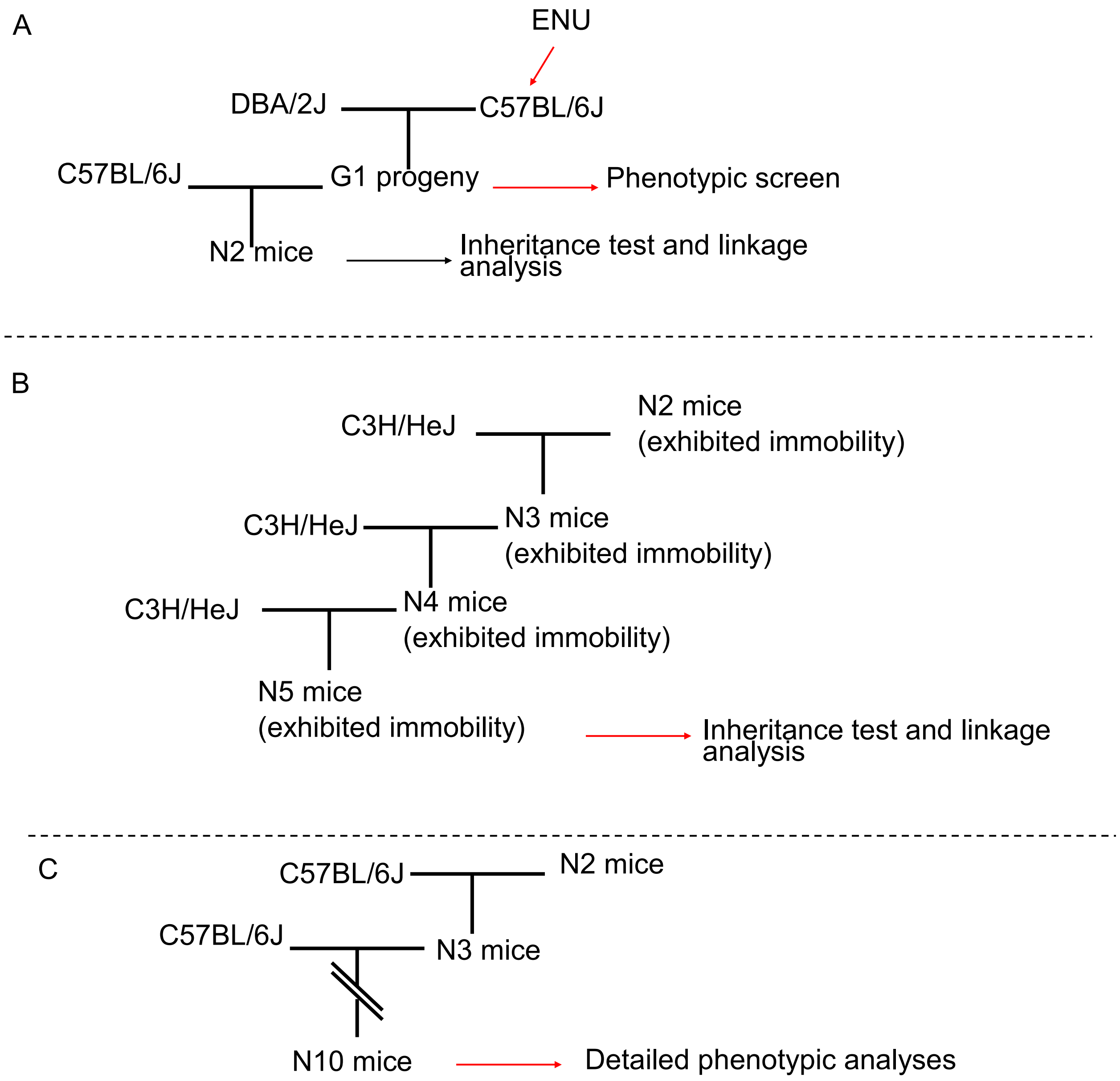

Figure S1. Strategy of animal production for phenotypic screen, genetic analyses, and detailed phenotypic analyses in ENU mutagenesis program

(A) Production of G1 mice for phenotypic screen.

B6 males were treated with ENU by intraperitoneal injection and crossed with D2 females. The progeny which was generated by this cross were designated as G1 mice. The G1 mice were used for phenotypic screens. The candidates for founder, phenodeviants, were crossed with B6 in order to generate N2 mice. N2 mice were phenotyped and genotyped for inheritance test and linkage analysis.

(B) Production of backcrossed progeny for linkage analysis

N2 mice of M100200 were crossed with C3 and N3 mice were generated. N3 mice were phenotyped for the passage production. The N3 mice that exhibited immobility were mated with C3 mice. The N4 mice and N5 mice were phenotyped for immobility and genotyped for SNP and microsatellite markers in order to map the causative mutation.

(C) Animal production for phenotypic analyses of M100200 mutant

The N2 mice was backcrossed to B6 at least 9 times (N10 mice) to eliminate genetic background of D2. The backcrossed progeny were used for all behavioral tests, biochemical tests, EEG recording and other phenotypic analyses.

Abbreviations, B6: C57BL/6J, D2: DBA/2J, C3: C3H/HeJ, ENU: N-ethyl-N-nitrosourea

Disease Models & Mechanisms • Supplementary information

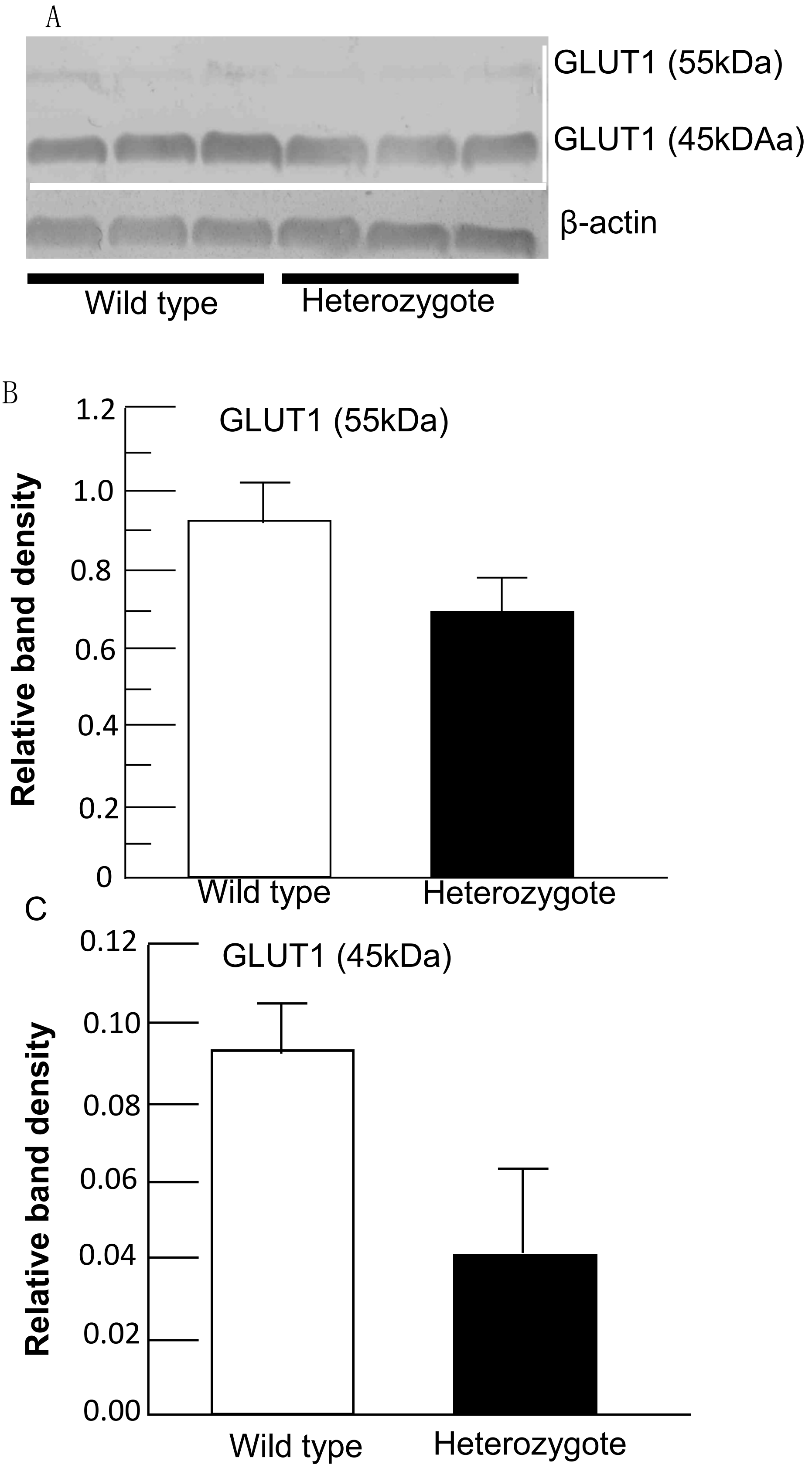

Figure S2. Immunoblotting of GLUT1 protein

(A) Protein fractions prepared from the fore brain of adult wild type and heterozygote of *Glut1*^{Rgsc200} were subjected to immunoblotting. A representative immunoblot for GLUT1 and d an internal control (beta-actin) is shown. The two Glut-1 isoforms (55 kDa and 45 kDa) and beta-actin (42 kDa) are indicated by arrow heads.

(B, C) Quantification of GLUT1 protein expression. The average expression level of two isoforms of GLUT1 relative to beta-actin is shown. There were no significant differences between the levels of expression in wild type and heterozygote. Error bars represent the S. E. M. Male mice, n = 3 of each genotype. Student's t-test, 55kDa GLUT1: $t_4 = 2.081$, $P > 0.1$; 45kDa GLUT1: $t_4 = 2.011$, $P > 0.1$.

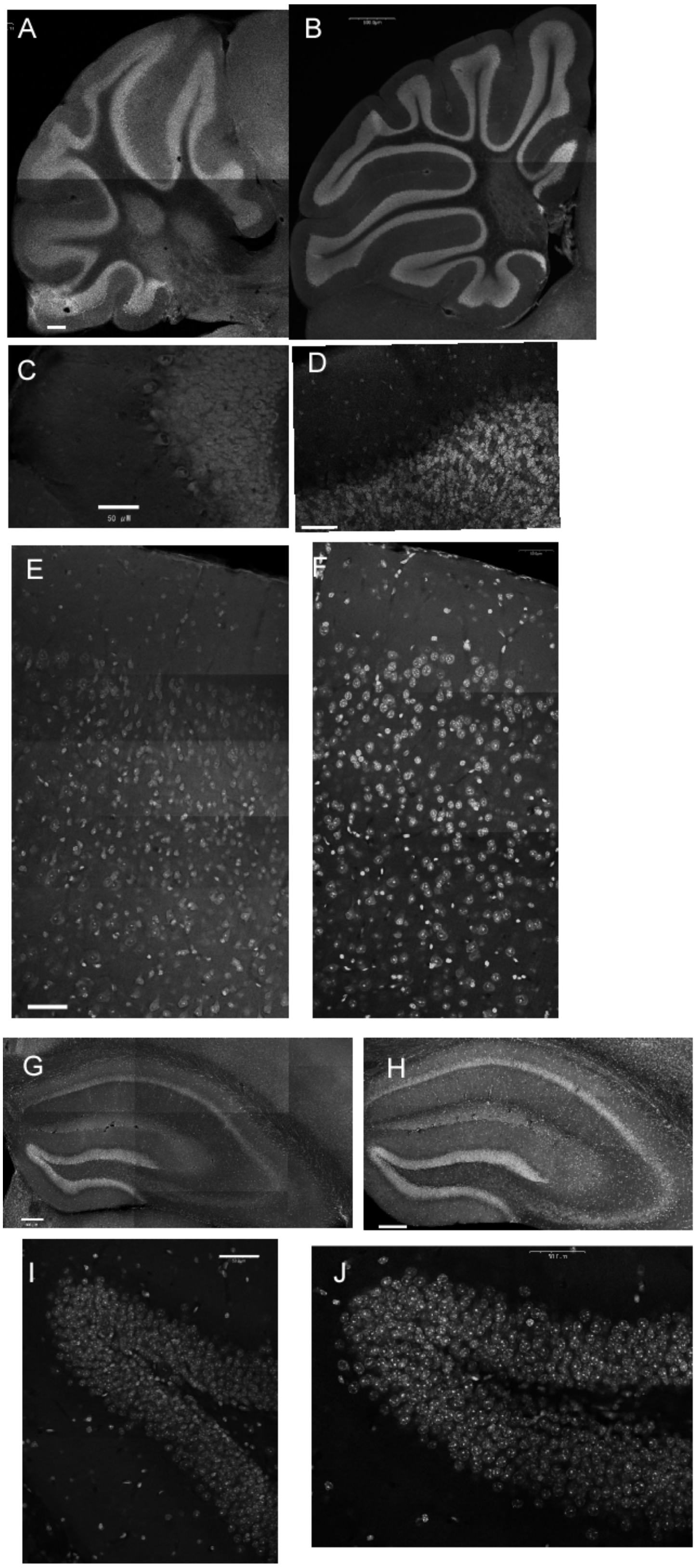

Figure S3. Brain histology of wild type and heterozygote of M100200 mutant

(A, B) Parasagittal sections of the cerebellum. (C, D) Higher magnification of the cerebellar cortex. (E, F) Coronal sections of the hippocampal cortex. (G, H) Coronal sections hippocampus. (I, J) Coronal sections of dentate gyrus. (A), (C), (E), (G), (I), and (K) are sections from wild-type mice whereas (B), (D), (F), (H), and (J) are sections from heterozygote. Scale bars: (A, B) 200 μm, (C, D) 50 μm, (E, F) 100 μm, (G, H) 200 μm, (I, J) 50 μm.

Disease Models & Mechanisms • Supplementary information

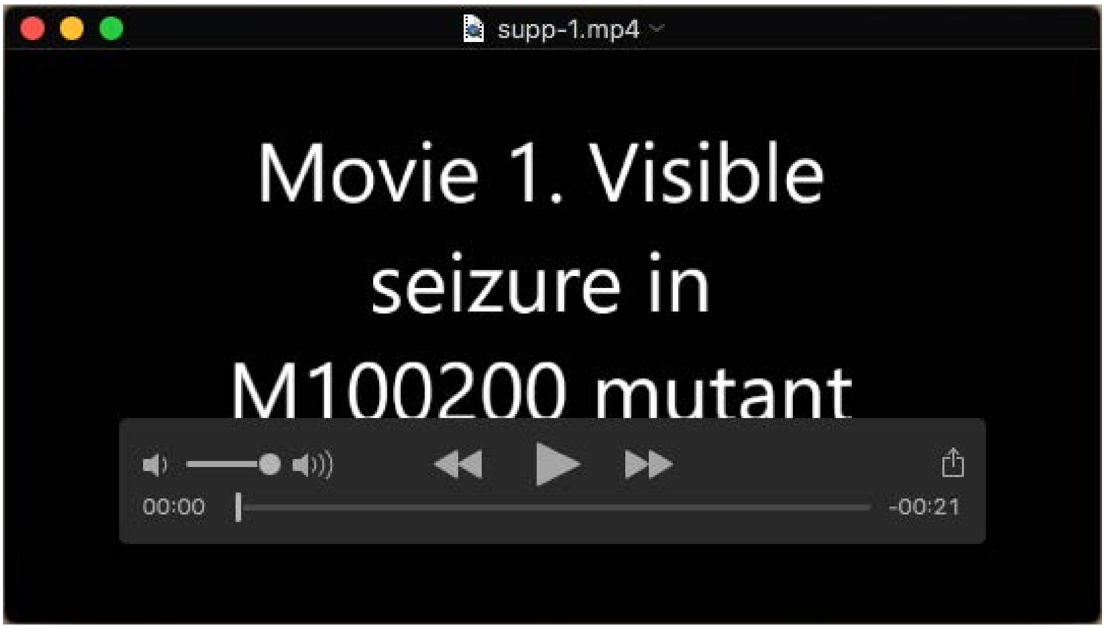

Movie 1. Visible seizures observed in the M100200 mutant. Immobility and convulsive seizure started suddenly with vocalization, ataxic gait, lowered posture, and slow movement. When the mutants are immobile, they continuously open their eyes and sometimes move their vibrissae.
